# Insights to Heart Development and Cardiac Disease Models Using Pluripotent Stem Cell Derived 3D Organoids

**DOI:** 10.3389/fcell.2021.788955

**Published:** 2021-12-02

**Authors:** Jeremy Kah Sheng Pang, Beatrice Xuan Ho, Woon-Khiong Chan, Boon-Seng Soh

**Affiliations:** ^1^ Disease Modeling and Therapeutics Laboratory, A*STAR Institute of Molecular and Cell Biology, Singapore, Singapore; ^2^ Department of Biological Sciences, National University of Singapore, Singapore, Singapore

**Keywords:** stem cell, embryonic development, organoid systems, cardiovascular disease modelling, drug screening, biomaterials

## Abstract

Medical research in the recent years has achieved significant progress due to the increasing prominence of organoid technology. Various developed tissue organoids bridge the limitations of conventional 2D cell culture and animal models by recapitulating *in vivo* cellular complexity. Current 3D cardiac organoid cultures have shown their utility in modelling key developmental hallmarks of heart organogenesis, but the complexity of the organ demands a more versatile model that can investigate more fundamental parameters, such as structure, organization and compartmentalization of a functioning heart. This review will cover the prominence of cardiac organoids in recent research, unpack current *in vitro* 3D models of the developing heart and look into the prospect of developing physiologically appropriate cardiac organoids with translational applicability. In addition, we discuss some of the limitations of existing cardiac organoid models in modelling embryonic development of the heart and manifestation of cardiac diseases.

## 1 Introduction

Congenital heart defects (CHD) affects approximately 0.4–5% of live births worldwide, making it the most common form of congenital disease (Kloesel et al., 2016). Despite advances to understand the molecular basis of CHD, limited knowledge of the aetiology is known. Despite use of complex animal models, such as pigs and mice to model CHD, species-to-species variability limits their translational potential. Hence, new models of CHD are required to improve our understanding of the conditions and explore potential therapeutic interventions.

The advent of pluripotent stem cells (PSCs), of both embryonic stem cells (ESC) and induced pluripotent stem cell (iPSC) origins differentiated into various cell lineages, provides novel insights to embryonic development and regenerative biology (Murry and Keller 2008; Zhu and Huangfu 2013; Tabar and Studer 2014). Human iPSCs offer a unique platform to study genetic mutations and developmental pathways associated with CHD. These iPSCs enabled advancements in cardiac embryogenesis research and helped uncover the critical roles morphogens such as BMP, Wnt, FGFs, TBX5 and GATA4 play in gastrulation and mesodermal patterning *in vivo* (Andersen et al., 2018). These finding have enabled the understanding of how diverse lineages and anatomical structure of the heart arise from the specification of two cardiac progenitor origins, the first heart field (FHF) and the second heart field (SHF).

More recently, organoids, characterized as three-dimensional (3D) *in vitro* tissue cultures have been widely used in multiple research fields to model their *in vivo* organ counterparts. Self-organized from stem cell populations, these organoids have the potency to undergo multiple divisions and differentiate into various appropriate cell types to confer structural and cell composition resembling *in vivo* organs (Lancaster and Knoblich 2014; Yin et al., 2016). Kidney, brain, intestinal, lung, retinal organoids amongst others have been used to study organ development as well as the effect of genetic modifications, disease onset and drug efficacy and toxicity, further enabled by the development of reprogramming techniques and CRISPR (Takahashi et al., 2007; Sun and Ding 2017). Cell-cell and cell-matrix cues presented within 3D organoids enable the differentiation, migration and self-organization properties of PSCs, overcoming the limitations of 2D monolayer cultures (Langhans 2018). As such, organoids hold the promise to revolutionize research on embryonic development, onset of diseases and drug development, thereby reducing our dependence on expensive animal and *in vivo* models. This review will focus on some key aspects of embryonic development of the heart, using 2D iPSC derived cardiomyocytes and 3D cardiac organoid models that serve to enhance our understanding of the genetic basis of development and pathophysiology of heart diseases. We also highlight some of the potential applications of existing cardiac organoid systems in modelling development, cardiac associated diseases and drug screening *in vitro*. Lastly, we discuss some of the key limitations of current *in vitro* models.

## 2 Current 3D Tissue Models of the *In Vivo* Heart

**FIGURE 1:**
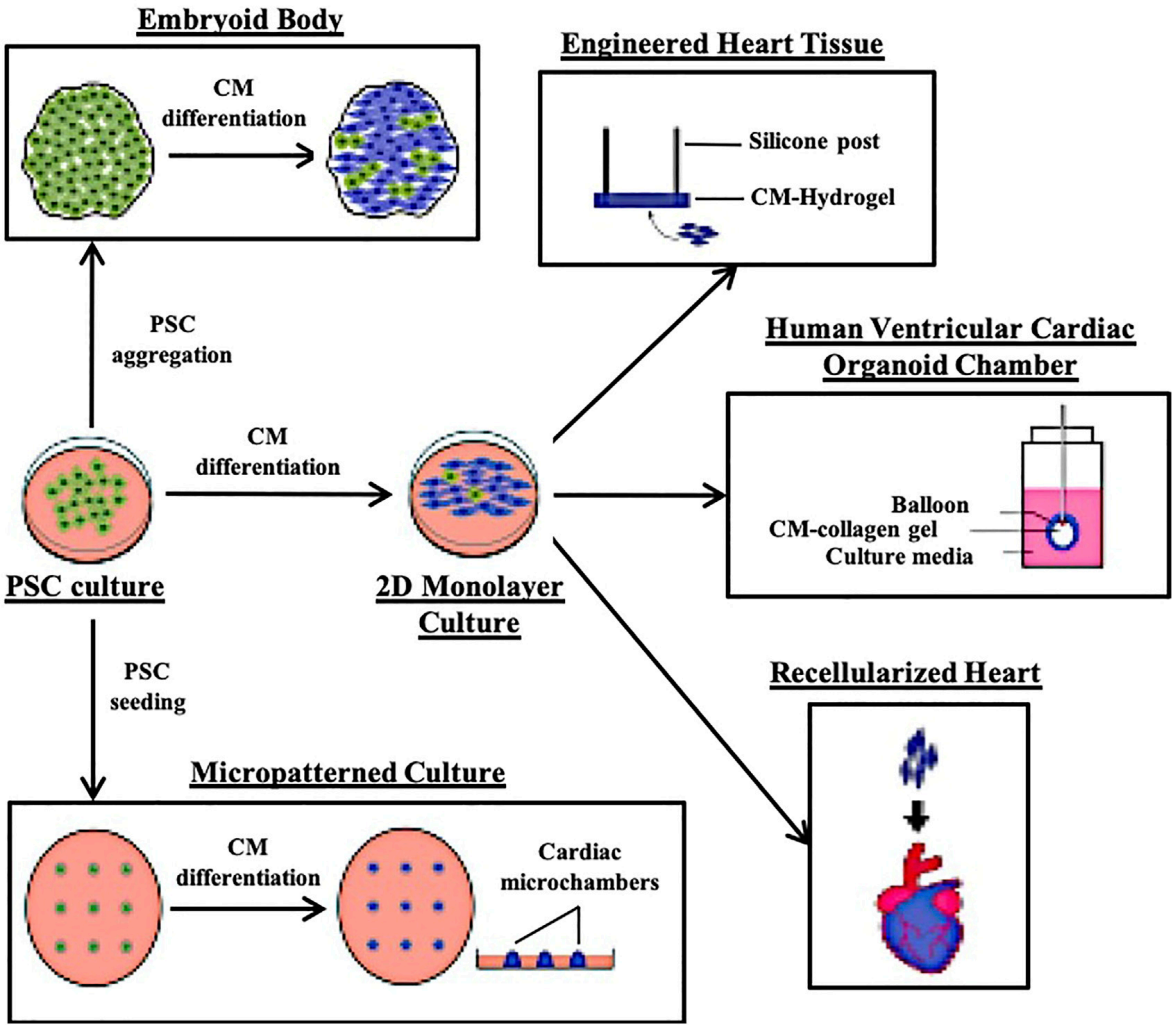
Illustrations of the different 3D cardiac modelling platforms that have been reported.

The human heart is highly complex, with a mixed population of cardiomyocytes (CM), epicardial cells, endocardial cells, smooth muscle cells, cardiac fibroblasts (CF) and endothelial cells (EC) with compositions that can vary with age or due to myocardial damage and remodelling (van den Berg et al., 2015). PSC-derived CMs (PSC-CMs) alone often exhibit immature metabolic capabilities, electrophysiological characteristics, sarcomere organization and contraction force compared with adult CMs (van den Berg et al., 2015). An *in vitro* model that can capture such a complex system, maintain, and mature the various cell types will be even harder to achieve. While cardiac organoid technology is still in its infancy, a number of research groups have developed methods to generate 3D cardiovascular tissue using either a pure CM starting population or a mixture of CMs, CFs and ECs and aggregating them into Engineered Heart Tissues (EHT) or on scaffolds (Figure 1) (Eschenhagen et al., 1997; Kraehenbuehl et al., 2008; Lee et al., 2008; Morizane et al., 2015; Polonchuk et al., 2017). A summary of recent cardiac systems adapted to provide biophysical, bioelectrical and biochemical cues to model cardiac related diseases and developmental defects are as summarized in Table 1.

**TABLE 1:** List of heart conditions modelled using various 3D cardiac models.

Condition	Model	Parameters assessed	Loci studied/Drug used
CPVT	EB	- Sarcomere alignment	*RYR2*, *CASQ2* (Novak et al., 2015)
		- Ca^2+^ signaling	
		- Contraction rhythm	
LQTS	EB	- Action potentials	*KCNH2* (Itzhaki et al., 2011)
		- Electrophysiology	
	EB	- Action potentials	*SCN5A* (Ma et al., 2013)
HCM	EB	- Cell size and structure	*MYH7* (Lancaster et al., 2013)
		- Multinucleation	
		- Transcriptome	
		- Electrophysiology	
		- Ca^2+^ signaling	
	EHT, Nanopatterned Monolayer	- Ca^2+^ signaling	*MYH7* (Yang et al., 2018)
		- Contractile force	
DCM	EB	- Apoptotic tendencies	*LMNA* (Siu et al., 2012)
ARVD/C	EB	- Ca^2+^ signaling	*PKP2* (Kim et al., 2013)
		- Bioenergetics	
		- Transcriptome	
AF	Atrial EHT	- Electrophysiology	Verankalant (Goldfracht et al., 2020)
		- Action potentials	
		- Conduction velocity	
CHD	Developmental organoid	- Cardiomyocyte compaction	*NKX2.5* (Drakhlis et al., 2021)
		- Cardiomyocyte and organoid size	
		- Transcriptome	
		- Contractility	
PGD	Developmental organoid	- Organoid size	Diabetic-like media (Lewis-Israeli et al., 2021)
		- Electrophysiology	
		- Bioenergetics	
		- Gene expression analysis	
		- Morphological organization	
Cardiotoxicity	Microtissues, Heart-on-chip	- Cardiomyocyte morphology	Sotalol, Verapamil (Visone et al., 2021)
		- Electrophysiology	
		- Interbeat variability	
		- Field potential duration	
		- Gene expression analysis	

CPVT, catecholaminergic polymorphic ventricular tachycardia; LQTS, Long QT syndrome; HCM, hypertrophic cardiomyopathy; DCM, dilated cardiomyopathy; ARVD/C, arrhythmogenic right ventricular dysplasia/cardiomyopathy; AF, atrial fibrillation; PGD, pregestational diabetes.

### 2.1 Embryoid Body Derived CMs, Spheroids and Organoids

Embryoid bodies (EBs) are aggregate masses of PSCs cultured in suspension and are widely used for differentiation into various tissue lineages. CMs were first derived using EBs generated using murine stem cells (Maltsev et al., 1993). Since then, various optimizations have been made to reliably generate contracting EB-CMs from PSCs. However, the main shortcoming of PSC derived disease models using the EB-CM approach is immaturity. The immaturity manifests in phenotypes such as smaller cell sizes, differentially expressed electrophysiological and cytoskeletal genes, lower resting membrane potential and weaker spontaneous contractions (Mummery et al., 2003; Kang et al., 2012; Yang et al., 2014). In other PSC derived tissue systems, maturation has been achieved by either transplantation into animal hosts or co-culture with additional cell types to supply biochemical cues in the form of paracrine signaling (Takebe et al., 2013; Finkbeiner et al., 2015; Dye et al., 2016; Asai et al., 2017; van den Berg et al., 2018). EB-CMs do contain tissue specific microenvironments and therefore CMs cultured within achieve some maturation and have been used as material for other 3D CM tissue models (Burridge et al., 2007; Zhang et al., 2009; Lee et al., 2011; Schaaf et al., 2011; Pesl et al., 2014; Mannhardt et al., 2016) or for disease modelling (Table 1).

To overcome the stochastic nature of EB-CM models resulting in widely varied cell compositions and cell number, cardiac spheroids have been aggregated using specific cell composition and number. A number of research groups have explored aggregating tissues of varying compositions appropriate to study specific archetypes of cardiac developmental diseases or health. Hanging drop culture has been used to aggregate CMs, ECs and CFs in a 3:1:6 ratio for spheroids to measure the cardiotoxicity of doxorubicin, a widely used cancer drug (Cao and Poss 2018). Another group showed that fusing CM and CF spheroids resulted in the migration and self-assembly of CMs and CFs within, giving rise to the intermixed morphology found in healthy myocardium (Polonchuk et al., 2017). Giacomelli et al. (2017) were able to generate cardiac microtissues by co-culturing purified CMs and ECs at an 85–15% ratio in a restricted culturing space, and show improved maturation and response to drug stimulation. They later show that these microtissues can also be formed including CFs, highlighting the microtissues utility in modelling cell-type specific diseases (Campostrini et al., 2021). These multi-lineage spheroid systems are highly applicable for cardiotoxicity drug screens and disease modelling due to their controlled nature leading to high reproducibility.

On the other hand, cardiac organoids are more strictly defined as 3D cardiac structures that were self-organized from either PSCs or multipotent progenitors. This direction is highly popular of late, with many recent works on utilizing either murine and human PSCs to generate cardiac organoids attempting to recapitulate cardiogenesis (Lee et al., 2020; Drakhlis et al., 2021; Lewis-Israeli et al., 2021; Rossi et al., 2021). Interestingly, work by Drakhlis et al. utilized lineage tracing to highlight extensive self-organization in their heart-forming organoid (HFOs) derived using an EB platform, which typically struggles to exhibit meaningful organization. HFOs exhibit many similar aspects of heart morphogenesis to the *in vivo* early heart and foregut endodermal anlagen of the developing embryo, with layered myocardium and endocardium, vascular network, and anterior-posterior foregut patterning (Drakhlis et al., 2021). By nature of its self-organization properties, these organoids are useful developmental models, but have not been widely adopted for disease modelling.

A popular method to overcome the maturation limitation of these spheroid and organoid models is long-term culture within bioreactors. Bioreactors supply biochemical signals by improving media circulation around the organoids to ensure higher mass transfer, physiological relevant gradient of nutrients and sufficient removal of waste products (Van Winkle et al., 2012). In addition, the flow of media around the organoids induce shearing forces which has been shown to act as a mechanical stretching stimuli, promoting CM maturation (Phelan et al., 2018). Therefore, long term bioreactor culture can potentially improve the maturation status of organoids to match that of adult heart tissue. However, the most prominent advances in bioreactor culture of PSC-CMs lie in the use of engineered heart tissues.

### 2.2 Engineered Heart Tissues

Engineered heart tissues are amongst the oldest and most utilized 3D tissue construct made up of aligned CMs with the ability to propagate contractions along a plane (Eschenhagen et al., 1997; Zimmermann et al., 2002; Schaaf et al., 2011; Mannhardt et al., 2016; Voges et al., 2017; Lemme et al., 2018). While the concept of EHTs was originally proven using animal CMs, human PSC-CM EHTs have been used for pharmacological studies (Schaaf et al., 2011). EHT constructs utilize hydrogels to cast PSC-CMs into molds with supporting post structures to align them in their longitudinal orientation. In the typical EHT setup, CMs aligned along two silicone posts can be subjected to mechanical tensile load and electrical stimulation while being simultaneously assessed for contraction forces and spontaneous rhythm (Hirt et al., 2012; Mannhardt et al., 2016; Yang et al., 2018). Tensile forces applied to the aligned CMs simulate hemodynamic load, and EHTs take advantage of synergistic electrical and mechanical stimulation to enhance CM maturation for extended durations (Hove et al., 2003; Reckova et al., 2003). Increasing the intensity of the tensile forces over time further enhances maturation as PSC-CMs were shown to exhibit adult-like gene profiles, organized ultrastructures, sarcomeric lengths, mitochondrial density, transverse tubules, oxidative metabolism, positive force-frequency relationship and functional calcium handling (Ronaldson-Bouchard. 2018). Bioreactors have been adapted to provide a cardiac biomimetic environment by perfusing media and biochemical factors while also providing paced electrical stimulation and mechanical stretching (Hirt et al.,2014; Hülsmann et al., 2017). EHTs in bioreactors with chemical, electrical, and mechanical stimuli exhibit better interconnectivity and alignment of CMs, stronger contractile forces and increased expression of cardiac-specificity proteins (Lu et al., 2012; Maidhof et al., 2012; Morgan and Black 2014; Visone et al., 2018).

EHTs have also been adapted to contain the appropriate cell composition for modelling specific diseases. ECs and/or CFs have been aggregated to study neovascularization and cardiac fibrosis (Caspi et al., 2007; Gabisonia et al., 2019). Purified atrial or ventricular cardiomyocytes have also been used to generate EHTs, which exhibit distinct atrial versus ventricular molecular and functional phenotypes, to be used for chamber-specific disease modelling and drug testing (Goldfracht et al., 2020).

However, there are limitations to EHTs as a modelling platform. It requires a high initial cell count for aggregation, within a complex equipment setup that prevents high throughput screens (Schaaf et al., 2011; Eder et al., 2016; Mannhardt et al., 2016). In addition, drug response studies suggest a limit to EHT achieved maturation (Turnbull et al., 2013). Therefore, whilst EHTs are a useful 3D platform to model contraction force as a parameter that cannot be easily quantified in monolayer CMs, the data generated may not be fully physiologically relevant for studies on the adult myocardium.

### 2.3 Micropatterned CM Cultures

EHT constructs of 3D CM tissues was popularized alongside several other hydrogel/polymer based matrixes for patterning (Camelliti et al., 2006; Khademhosseini et al., 2007; Cimetta et al., 2009; Kim et al., 2010; Morizane et al., 2015). Conceptually, the idea of micropatterning is similar to EHTs in that the geometric space for cell population is restricted to encourage cell organization and alignment. There are diverse protocols of generating these patterned cultures, each suited to the requirements for the desired treatments and measurements needed. It is out of the scope of this review to introduce and address the pros and cons of these micropatterned protocols for cardiac disease modelling, as micropatterned cultures are mostly 2D and do not sufficiently recapitulate the architecture of a 3D human heart. However, one research group has developed a cardiac differentiation protocol of micropatterned human iPSCs to generate a self-organized, 3D cardiac microchamber with a developing heart phenotype. They also demonstrated microchamber utility by modelling a cardiac developmental defect due to thalidomide treatment, a known chemical that causes cardiac birth defects (Morizane et al., 2015). While useful to model developmental defects, the cardiac microchamber, like the other 3D models discussed above, is not adapted for modelling of the adult heart.

### 2.4 Scaffold-Based CM Cultures

Similarly, hydrogels and polymers have been utilized to create 3D scaffolds as a biomimetic approach to recapitulate human cardiac tissue (Kraehenbuehl et al., 2008; Grayson et al., 2009; Maidhof et al., 2012). While EB and EHT models listed above are 3D cardiac cultures, they are not more environmentally controlled as their monolayer counterparts. Another crucial need of cardiac tissue research is a controlled and replicable differentiation program for regenerative medicine, such as tissue transplants for hearts damaged by myocardial infarction. Specific organ-derived decellularized scaffolds have been shown to retain defining features of the organs and contain functionally and structurally relevant molecules such as growth factors, fibronectin and collagen, which all play a part in directing cell distribution, organization and attachment within the scaffold (Brown et al., 2006; Badylak et al., 2011). In the cardiac field, human CM progenitors were shown to repopulate a mouse decellularized heart scaffold by spontaneous migration, proliferation and differentiation. These repopulated hearts exhibited measurable electrophysiological and mechanical properties and responded appropriately to chemical treatments to induce tachycardia and arrhythmia (Lu et al., 2013). However, as with most organ research, obtaining human organs for use is difficult and would exacerbate the world organ shortage issue. On the other hand, there are a handful of protocols developed to synthetically create or bioprint 3D scaffold structures, which utility is currently being looked into for generating 3D organized CMs (Kraehenbuehl et al., 2008; Hockada et al., 2012; Gaetani et al., 2015; Zhang et al., 2016; Ong et al., 2017).

While cardiomimetic scaffolds have been developed to provide a geometrically and environmentally appropriate 3D architecture for CM seeding, simple folded scaffolds have also been developed to promote cardiac chamber formation (Lee et al., 2008; Li et al., 2018). These human ventricle-like cardiac organoid chamber termed by Li et al. were generated by immersing a balloon core into an EB-CM suspension for CMs to aggregate on the balloon surface, essentially serving as a curved surface scaffold for a cardiac chamber. Eventually, the balloon core is removed and a hollow chamber with CMs forming the chamber wall remains. This surface scaffold set-up is indeed useful as the hollow chambers are unique and allows ejection fraction and pressure to be monitored. However, these hollow chambers might not truly model the adult heart chambers as it is no more than an aggregation of heterogeneous EB-CMs on a curved 2D surface, and not a result of self-organized CMs.

Alternatively, precise control of cellular composition and spatial distribution of individual cells can be achieved via 3D bioprinting. Hydrogels and decellularized extracellular matrix have been developed into bioinks that serve as a scaffold for individual cell types to be printed on (Lepilina et al., 2006; Caspi et al., 2007; Sekine et al., 2008; Weeke-Klimp et al., 2010). Of note, a research group recently utilized 3D bioprinting with personalized hydrogels to construct cardiac patches matching the patient’s myocardium. They also printed out a whole miniature heart that contained the major blood vessels (Zhou and Pu 2008). While these approaches allow for highly controlled generation of cardiac constructs, they tend to be expensive, not readily scalable and do not accurately capture developmental cues present for embryonic heart development.

## 3 Recapitulating Cardiogenesis Using Cardiac Organoid Models

Congenital heart defects occurring during the first 8 weeks of embryonic development are detrimental to the growing embryo’s ever-increasing metabolic demands during embryogenesis. However, inaccuracies in the *in vitro* heart models limit research in understanding these disorders. While much effort has been poured into generating the 3D CM models described in Section 2, not all are applicable for studying developmental pathways. In particular, models populated with externally derived CMs such as the spheroids, EHTs or scaffold-based systems cannot recapitulate human cardiogenesis. Organoids on the other hand, exhibit self-organization and patterning properties and are thus of interest for studying these earlier developmental processes. An extensive review that characterizes current organoid models and their required developmental morphogens was recently published by Miyamoto et al. (2021), offering an interesting perspective on the importance of organogenesis aligned with *in vivo* development.

There have been significant recent advances in the field of developmental cardiac organoids, which were intrinsically formed by taking advantage of the self-organizing and patterning capabilities of either murine or human hPSCs. These *in vitro* models were shown to recapitulate various aspects of cardiogenesis. Earlier work by Soh et al. (2016), demonstrated the critical role of WNT3A in expansion of the SHF progenitors, resulting in the intrinsic ability of vascular progenitors to develop and self-organise into cardiac tissues. Similarly, Andersen et al. (2018), provided insights to the critical role of BMP/WNT signalling into heart field specification using both mouse and human PSCs, which may be leveraged for modelling developmental defects during gastrulation. This study recapitulated early stages of heart field development, based on the formation of two distinct heart fields using mouse ESCs differentiated into precardiac organoids.

Consistently, Rossi et al. (2021) generated axially patterned organoids termed gastruloids using mouse ESCs with the capability of supporting cardiovascular progenitor FHF and SHF cells. These gastruloids exhibited spatially organized structure, mimicking the development of cardiac crescent like structures, and formed contracting cardiac tissues near a putative primitive gut-like tube separated by an endocardial layer. Thus, this platform highlights the ability of mouse ESCs to coordinate the organization potential of multiple tissue types to model heart development at an unprecedented detail.

Developmental organoids can also possess the capability to be used as a disease modelling tool, particularly for drug testing. Heart organoids were generated using mouse ESCs by utilizing FGF4 and extracellular matrix, emulating the developmental processes of the *in vivo* heart (Lee et al., 2020). These heart organoids would therefore be of a later developmental stage than the gastruloid model, as they possess atrium- and ventricle-like structures, conducting tissues, smooth muscle and endothelial cells organized similarly to a mammalian chambered heart. The authors highlighted that the ECM microenvironment plays the key role to induce *in vitro* organ/tissue formation to mimic cardiogenesis from the early to mid-gestation stages, and thus does not require complex differentiation protocol. Therefore, the heart organoids represent a promising research tool to study developmental diseases for drug testing.

### 3.1 Cardiac Organoids for Disease Modelling and Drug Screening

As mentioned, several organoid platforms have focused particularly on the disease modelling and drug screening purposes of the model (Spence et al., 2011; Mae et al., 2013; Xia et al., 2013). These modelling organoids have been used to mimic embryonic development, but have also been used for extensive maturation, regenerative medicine and studying chamber specific diseases. Table 2 summarizes a list of increasingly popular organoid models for disease modelling.

**TABLE 2:** Summary of recent developed organoid systems and their contribution to cardiogenesis and disease modelling research.

Applications	Model	Observed phenotype	References
Development	Human PSCs	Heart organoids exhibited comparable transcriptomic, structural, and cellular level as age-matched human fetal cardiac tissues	Lewis-Israeli et al. (2021)
	Human PSCs	Cardiac organoids mimics human early heart and foregut development	Drakhlis et al. (2021)
	Mouse and human PSCs	Critical role of BMP/WNT signalling in the formation of two heart fields, such as FHF and SHF in precardiac organoids	Andersen et al. (2018)
	Mouse ESCs	Critical role FGF4 and extracellular matrix for the formation of functional murine heart organoids	Lee et al. (2020)
	Mouse ESCs	Gastruloids supports differentiation of cardiac progenitors, such as FHF and SHF	Rossi et al. (2021)
	Human PSCs	Chamber-like cardioids recapitulates heart lineage architecture to specify, pattern, and morph into a cavity *in vitro*	Hofbauer et al. (2021)
	Human PSCs	Development of a 96-well platform to functionally screen human cardiac organoids to enhance maturation of hPSC-CMs	Mills et al. (2017)
	Human PSCs	Generation of spatial-patterned early developing 3D cardiac microchambers using a combination of biomaterials-based cell patterning with stem cell organoid engineering	Hoang et al. (2018)
CHD	Human PSCs	Modelling pregestational diabetes-induced congenital heart defects	Lewis-Israeli et al. (2021)
Drug screening	Human PSCs	Bioengineered human cardiac organoids present a platform for identifying pro-regenerative drug compounds	Mills et al. (2019)
	Human PSCs	Micropatterned engineered spatially organized cardiac organoids for assessment of drug-induced developmental cardiac toxicity	Hoang et al. (2021)
Myocardial infarction	Human PSCs	Modelling of myocardial infarction and doxorubicin induced cardiotoxicity in cardiac organoids	Richards et al. (2020)
Regeneration	Human PSCs	Human cardiac organoids exhibited regenerative capacity towards cryoinjury, with full functional recovery after 2 weeks	Voges et al. (2017)

Of note, Lewis-Israeli et al. (2021) recently generated self-assembling human heart organoids (hHOs) differentiated *via* Wnt signaling modulation, and extensively detailed its capability in modelling cardiac development and diabetes-induced congenital heart disease. These hHOs were mainly comprised of myocardial tissue, with epicardial tissue organized near the exterior surface of the organoids. In addition, these hHOs recapitulate the functional and structural features of the developing fetal heart, exhibiting robust contraction and action potential waves reminiscent of the PQRST waves, and expressed well defined sarcomeres surrounded by gap junctions, mitochondria and t-tubules. As a proof of concept, pregestational diabetes impacted the cardiac development of these hHOs, resulting in irregular arrhythmic contraction, metabolic dysfunction and structural organization in accordance with the phenotype observed *in vivo*.

Myocardial infarction and drug cardiotoxicity has been recently modelled in a cardiac microtissue organoid model as well (Richards, 2020). The unique property of organoids being a 3D model allows for accurate modelling of diffusion gradients, which is particularly useful for an infarction model. Richards et al. utilized an oxygen-diffusion gradient with noradrenaline to mimic the zoning aspects of infarction, and then recapitulating aspects of infarction at the transcriptomic, structural and functional levels. The authors also demonstrated image-based functional analysis to screen for drug cardiotoxicity.

## 4 Limitations of PSC-Derived Cardiac Organoids for Modelling Congenital Heart Defects and Heart Diseases

Collectively, developments in 3D cardiovascular disease models have demonstrated the ability of patient-derived cardiac organoids to recapitulate developmental processes of cardiogenesis, as shown by ultrastructural, gene expression, histochemical characteristics with resemblance to the developing heart *in vivo*. However, current cardiac organoid models fall short in developing important structural and morphological elements unique to the heart. Particularly, cardiac architecture, most notably being the heart chambers are absent, and thus related elements such as the septa and valves are also missing. As such, CHD affecting these missing elements in the *in vivo* developing heart cannot be accurately modelled.

Cardiac progenitors within the embryonic heart specifies their subtype early in development, primarily during heart field specification (Lescroart et al., 2014). However, current cardiac organoid models do not recapitulate this specification event and fail to develop organization at the level of chamber subtypes. This limitation is due to current differentiation protocols favoring the generation of ventricular CMs, with small populations of atrial cells mixed in Lian et al. (2012). To enrich for atrial specification, recent differentiation protocols have incorporated retinoic acid (Zhang et al., 2011). As the methodology to encourage self-organization and specification into chamber specific organoids have not been discovered, the current workaround is for researchers to generate and purify atrial or ventricle specific CMs to model chamber specific diseases (Lemme et al., 2018; Zhao et al., 2019; Goldfracht et al., 2020). Taking a step towards overcoming this limitation, recent work demonstrated that cardiac spheroids could be generated using FHF or SHF cell populations, which developmentally contribute to the different heart chambers *in vivo* (Andersen et al., 2018). Further advances into developing chamber specific organoid models would be required for in-depth modelling of heart field related or chamber-specific CHD.

Lastly, the missing structural elements of current cardiac models results in the failure to model mechanical functional changes such as myocardial wall thickness, pressure loading and ejection fraction. Heart disease often present with pathological cardiac remodeling, causing hypertrophic growth, irreversible decompensation and thinning of the myocardium and dilatation of the failing heart (Chien 1999; Hill and Olson., 2008). Classically, these mechanical parameters are assessed in animal models or echocardiography (Sasayama et al., 1976; Alpert et al., 1985). Cardiac organoids would be an ideal system to recapitulate the human heart *in vitro*, working in tandem with classical models for accurate phenotyping of disease and drug effects. However, the lack of functional cardiac chambers limits the utility of these organoids in the disease modelling space. The closest model in achieving self-organized chambers currently available is the hHO model by Lewis-Israeli et al., detailed previously in Section 3.1, which exhibit intrinsic development of multiple cardiac microchambers lined with NFATC1^+^ endocardial cells (Lewis-Israeli et al., 2021). However, functional disease modelling data utilizing the chamber contraction or wall thickness is still absent. An alternative would be the human ventricle-like cardiac organoid chamber by Li et al., which contains an artificially generated chamber by forced aggregation of CMs to line a hollow surface (Lee et al., 2008; Li et al., 2018). Such an artificially generated platform would be the closest system to assess cardiac ejection fraction, in the absence of a fully self-organized cardiac chamber model.

## 5 Future Direction of PSC-Derived Cardiac Organoid Models

**FIGURE 2:**
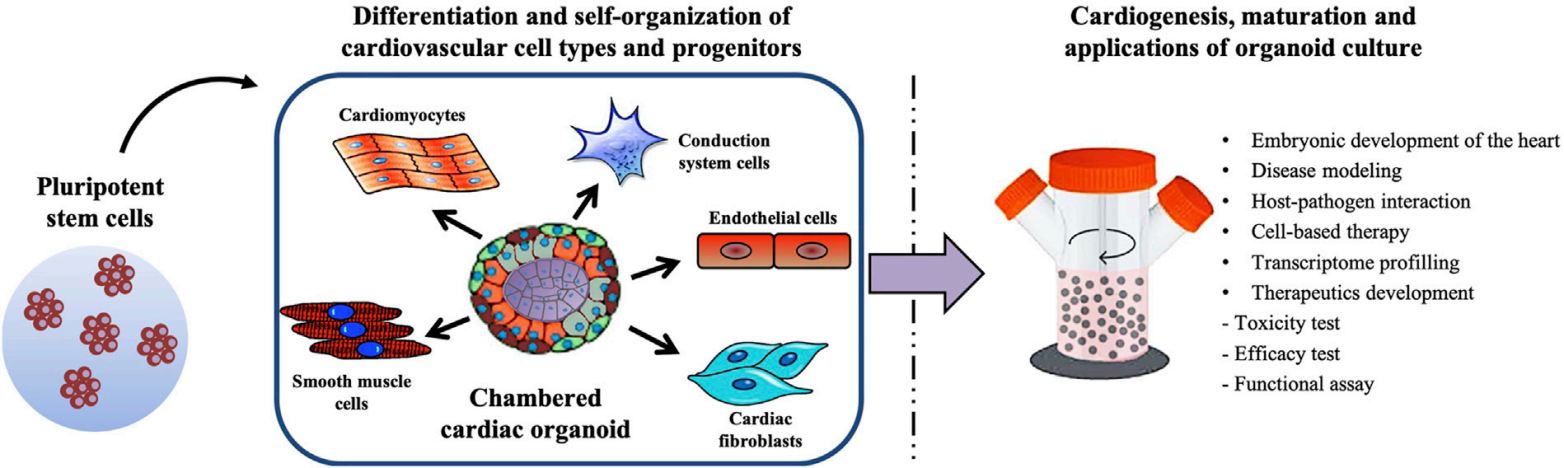
Schematic summarizing the generation of a multicellular chambered cardiac organoid, and its maturation process and applications.

Drawing from the approaches of the tissue organoid systems currently generated, intrinsically formed chambered cardiac organoids should theoretically be achievable. Judging by the rapid pace of advances in the space of cardiac organoid development in recent years, there is promise that such an organoid model will soon be developed and be scalable for high-throughput disease modelling. Figure 2 illustrates the possible methodology behind generating a physiologically representative chambered cardiac organoid—by allowing the self-organization of multiple cardiovascular cell types followed by long-term maturation in a bioreactor system. Adapting these theoretical cardiac organoids for high throughput disease modelling will require analysis platforms that can assess multiple facets of cardiac function in real time, including electrophysiological measurements, contractile mechanical forces and ejection fraction.

Lately, there has been increasing interest in utilizing microfluidic systems such as organ-on-a-chip platforms for use in preclinical analysis. These chip-based systems are the preferred platform for industry-level research—completely automated high throughput derived organ-models are screened within highly reproducible fabricated chips. Organ-chip systems have been adapted to allow for vascularization in a number of tissue types, recently reviewed by Osaki et al. (2018), which is an additional advantage over traditional cultures lacking vascularization. A number of heart-on-a-chip systems have been developed, utilizing either EHTs or cardiac microtissues (Grosberg et al., 2011; Wang et al., 2014; Marsano et al., 2016; Nawroth et al., 2018; Visone et al., 2021). These heart-on-a-chip platforms embody the necessary screening platform of the future, with the ability to supply simultaneous electrical and mechanical stimulation for maturation and long-term culture, while also probing for functional electrical and mechanical abnormalities. The next breakthrough of cardiac disease modelling will thus likely revolve around the development of cardiac organoid disease models utilized in high-throughput analysis microchips for accurate modelling of CHDs and cardiovascular diseases.

## 6 Conclusion

While 3D CM cultures currently exist and are routinely used for studying development, drug screening and disease modelling, they often portray an incomplete picture of associated effects on a human heart. The strong demand required to bridge the technology and knowledge gap will enable researchers to better study heart development and cardiovascular diseases for research and translational purposes. Hence, future direction for cardiac organoid research is currently focused on generating physiologically appropriate chambered cardiac organoids paired with an appropriate high-throughput modelling platform that can simultaneously assess multiple cardiac functional parameters. With more faithful models of the *in vivo* heart in place, future *in vitro* cardiac modelling will be able to better support downstream preclinical pharmaceutical research in discovering cures and preventing cardiotoxic drugs from slipping through.
